# Breathlessness from the Perspective of the Persian Medicine

**Published:** 2016

**Authors:** Ali Abdolahinia, Mohsen Naseri, Alireza Eslaminejad, Farzaneh Ghaffari, Ali Akbar Velayati

**Affiliations:** 1 Department of Iranian Traditional Medicine, Faculty of Medicine, Shahed University, Tehran, Iran; 2 Traditional Medicine Clinical Trial Research Center, Shahed University, Tehran, Iran; 3 Chronic Respiratory Disease Research Center, National Research Institute of Tuberculosis and Lung Disease (NRITLD), Shahid Beheshti University of Medical Sciences, Tehran, Iran; 4 School of Traditional Medicine, Shahid Beheshti University of Medical Sciences, Tehran, Iran

**Keywords:** Iranian traditional medicine, Persian medicine, Breathlessness, Dyspnea

## Abstract

Dyspnea is one of the most complaints in the pulmonary diseases. Shortness of breath as a subjective symptom can decrease the quality of life of patients. Although symptomatic treatment of the patients with chemical drugs is efficient in sign reduction, drugs side effect and allowing the disease to become chronic are risky for patients. Nowadays, traditional medicine is considered as an effective strategy in patients′ treatment by World Health Organization. This study discusses the causes of shortness of breath from the view of Iranian traditional medicine and describes some suggestion for treatment of causes of this problem.

Persian medicine prioritizes prevention of diseases by offering some strategies. In case of disease occurrence, life style modifications and herbal pharmaceutical therapy are recommended.

## INTRODUCTION

Difficulty in breathing described as feeling of “shortness of breath”, “cannot get enough air”, and “tightness in the chest” is referred to as breathlessness and the medical term for it is dyspnea (1).

Many pulmonary conditions such as pulmonary emboli, chronic obstructive pulmonary diseases, pneumonia and pulmonary hypertension as well as non-pulmonary conditions such as cardiac diseases, anemia, hernia, obesity and anxiety disorders can all cause breathlessness. Shortness of breath as a subjective symptom can accompany chest pain and dizziness and decrease the quality of life of patients. Resolving this symptom by treating the cause allows easier passage of air into the lungs and efficient gas exchange in the alveoli. Bronchodilators, anti-inflammatory drugs and corticosteroids can resolve this symptom (2). Iranian traditional medicine or Persian Medicine (PM) is based on temperament and prioritizes prevention by modifying lifestyle followed by treatment with medicinal herbs. Traditional medicine has several recommendations and strategies for treatment of shortness of breath based on the causative agent. This study discusses the causes of shortness of breath from the view of Iranian traditional medicine and describes the suggested strategies for treatment of causes of this problem.

### Respiration in the Persian Medicine

Avicenna (980–1037 AD), as the pioneer of Iranian traditional medicine, divided breathing into two movements and two stops. The difference between breathing and pulse is in that breathing is voluntary. He also described different modes of breathing including large and small, deep and shallow, fast and mild, different and frequent, vast and narrow, easy and difficult, strong and weak, and warm and cold. Differentiation of these conditions is interesting considering the limitations in equipment at that time. Breathlessness from the perspective of the PM refers to abnormal breathing and in most cases, parts of the respiratory system are involved. However, breathlessness may be due to fullness, obstruction, proximity to compressive organs or masses, edemas or swellings, severe pain, wound in the chest or diaphragm, loss of strength (weakness), high fever and oral toxins (3).

### Factors Causing Shortness of Breath According to the Persian Medicine

1. Narrowing of airways: which may occur due to the swelling of larynx, trachea or bronchial tracts or presence of tumors or abscess in the respiratory tract. If the stricture is in the bronchi or bronchioles, it causes pain and burning sensation and leads to coughing. Fast shallow breathing accompanied by fever indicates swelling in the lungs or other parts of the respiratory system.
2. Presence of sputum in respiratory tracts: If breathing is fast and shallow but is not associated with fever, it may be due to the presence of sputum. Its location is found based on the presence/absence of cough.
3. Presence of mucus in internal body spaces such as pleural cavity, or presence of ascites can prevent expansion of diaphragm and cause shortness of breath.
4. Weakness or paralysis of the muscles of respiration contributing to inhalation and exhalation.
5. Air entrapment in the chest. (Pneumothorax)
6. Congenitally small size of the lungs and chest, preventing complete expansion of the lungs. These patients suffer from shortness of breath in their entire life.
7. Enlarged and stiffed liver: Enlarged, stiffed liver pressures the diaphragm and prevents its expansion.
8. Improper temperament such as dryness of chest and cold temper of the lungs (as seen in the elderly)
9. Trauma and injury to the brain
10. Bleeding in the respiratory system and blood clot in respiratory tract causing stimulation and aggravation of shortness of breath.
11. Presence of dust or smoke in the air
12. Impairment of the diaphragm nerve

Resolving of shortness of breath in each case requires elimination of the cause, which is different for each case. According to Avicenna, some problems in the stomach, liver, uterus and intestines can be responsible for development of shortness of breath. Thus, it is important to thoroughly examine all organs in patients suffering from shortness of breath.

### Treatment

The Iranian traditional medicine recommends three different strategies (in terms of priority):

#### Lifestyle modification

Patients suffering from shortness of breath must first modify their lifestyle. PM has some general recommendations for these patients, which covers all types of shortness of breath. These recommendations include air pollution avoidance, (4) nutritional advices such as consumption of fermented bread, with additives such as hyssop, thyme and pennyroyal. Small freshwater fish and small birds are suitable as protein sources for these patients. According to the PM, chickpea is among the main foods suitable for the lungs. However, flatulence must also be taken into account because avoiding flatulence caused by beans is another recommendation for prevention of shortness of breath. Addition of plantain leaf to meals, drinking grape juice and honey syrup have also been recommended for these patients. Food and drinks should not be used simultaneously and a time interval must be considered between them. Drinking of beverages must be slow, and drinking (water or other beverages) must be avoided after meals. Iranian traditional medicine also has specific recommendations for sleeping, bathing and physical activity of these patients. Sleeping shortly after eating should be avoided since it is highly hazardous for these patients unless the cause is exhaustion. In the latter case, a short nap is recommended. Patients with shortness of breath must avoid long baths especially after heavy meals or long sleep. Avicenna recommended special physical exercises for these patients. Singing (starting from a low tone and gradually raising the tone, power and length) is also helpful. Massage of the chest by thick fabrics with or without oil has also been recommended to eliminate shortness of breath in some patients (3). Prevention and treatment of constipation must be done prior to any other treatment for these patients.

#### Herbal Remedies

PM has several prescriptions for treatment of patients with shortness of breath. Herbal medications may be administered alone or in combination. They can be boiled or brewed. Avicenna recommended the use of lozenges for delivery of drug to the target site. In this method, drug is gradually released and reaches the target site due to the proximity of esophagus and respiratory tract (5). Of different medicinal plants used for this purpose, the below mentioned herbal medicines play a significant role in treatment of shortness of breath: Table-1

**Table:** 

**Medicinal plant**	**Common name**	**Traditional name**	**Part used**	**Pharmacological effect**	**Temperament**
Hyssopus officinalis L.; Nepeta Bracteata Benth.	Hyssop	Zoofa	Flower	Anti-fungal, Anti-microbial	Hot and Dry
Adiantum capillus-veneris	Maidenhair fern	Parsiavoshan	Leaf		Hot and Dry
Glycyrrhiza glabra L.	Liquorice	Shirin-bayan	Root	Anti-microbial	Hot and Dry
Foeniculum vulgare L.	Fennel	Razianeh	Seed	Anti-microbial, Bronchodilator	Hot and Dry
Trigonella foenum-graecum	Fenugreek	Shanbelileh	Leaf, Seed	Mucolytic	Hot and Dry
Cydonia oblonga Miller	Quince	Safarjal, Beh	Fruit	Anti-allergic	Cold and Dry
Dorema ammoniacum D. Don.	Ammmoniacumm	Oshagh	Resin	Anti-microbial, Antifungal	Hot and Dry
Viola odorata L.	Sweet violet	Banafsheh	Flower	Anti-microbial	Cold and Wet
Althea officinalis L.	Althea	Khatmi	Flower	Anti-inflammatory	Temperate
Crocus sativus L.	Saffron	Za’feran	Flower		Hot and Dry

##### Hyssop (Hyssopus officinalis, Nepeta Bracteata Benth)

Hyssop is one of the famous medicinal herb used for treatment of pulmonary diseases and allergic rhinitis since the Hippocrates. Studies have shown that this plant is effective for treatment of inflammatory lung diseases and has anti-spasm, antiviral, antimicrobial (against Staphylococcus aureus and Escherichia coli) and antifungal (against Candida albicans) effects (6). Avicenna used this herbal medicine (Gol – e – Zoofa) as one of his most effective remedies for treatment of shortness of breath (3). It has been commonly prescribed for chronic cough, shortness of breath and rhinitis in the past 1000 years.

##### Maidenhair fern (Adiantum capillus-veneris)

Aerial parts and leaves of this plant are used for treatment of respiratory diseases (7). At present, this drug is used in cough medicines manually prepared by pharmacists (8). Razi in his famous book “Al Havi” states that “Maidenhair fern is a thin plant, which grows by the raceways and quickly loses its effect. It cleans the lungs from thick sputum” (9). Avicenna also believed that this medicinal plant is very effective for cleaning of the lungs and treatment of cough (3).

##### Liquorice (Glycyrrhiza glabra)

Although Liquorice is known as a gastrointestinal medication among people, animal studies have confirmed its efficacy for decreasing the degree of inflammation of lung parenchyma and pulmonary fibrosis (10). Also, Liquorice root is used for treatment of severe coughs. Avicenna recommended it for cleaning of respiratory tract and treatment of hoarseness.

##### Fennel (Foeniculum vulgare)

Romans believed that snakes use fennel to strengthen their vision. Antibacterial effects of fennel oil have been confirmed in pulmonary patients (11). Also, anti-inflammatory, antimicrobial and bronchodilator effects of fennel have been previously reported (12). Avicenna recommended addition of green fennel to breast milk to treat shortness of breath in infants (13).

##### Fenugreek (Trigonella foenum-graecum)

Recent studies have confirmed the efficacy of fenugreek for decreasing the symptoms of nasal congestion, sinusitis, cough and sputum production due to its mucolytic properties (14). Seeds of this plant are rich in proteins, lysine, tryptophan, saponin and diosgenin, and contain large amounts of soluble fibers such as hemicellulose, saponin, mucilage, tannin and pectin, which are used for treatment of diabetes mellitus and hypercholesterolemia (15).Razi recommended its cooked form for chronic chest pain without fever and its electuary for shortness of breath (9).Avicenna recommended it for hoarseness and stated that its dressing is suitable for pleurisy when applied on chest (3).

##### Quince (Cydonia oblonga Miller)

Its seed glaze is used as a remedy for cough in many parts of the world. Anti-allergic effects of its boiled extract have been previously confirmed as well and its leaves and seeds are used for treatment of bronchitis and chronic cough (16). Evidence supports the anti-oxidant and antimicrobial properties of some parts of this plant as well. However, toxic effects of chewing the seed must also be taken into account (17,18).

##### Ammmoniacumm (Dorema ammoniacum D. Don.)

Antibacterial and antifungal effects of ammmoniacumm products have been confirmed. Avicenna recommended its lozenge with honey or boiled barley for shortness of breath and also prescribed it for treatment of diaphragm wounds (19). Razi also used this plant in his remedies for shortness of breath and cleaning of the lungs from thick sputum. However, new evidence regarding pulmonary effects of this plant is scarce.

##### Althea (Althea officinalis)

All parts of this plant produce a lot of glaze. Its boiled flower is effective for treatment of common cold and fever and is very beneficial for dry coughs (20).

##### Saffron (Crocus sativus)

Avicenna discussed the effects of saffron on shortness of breath and stated that “saffron is highly beneficial for treatment of breathlessness due to its effect on respiratory tract” (3).

##### Sweet violet (Viola odorata)

Evidence shows that sweet violet can treat children’s cough (21). Jorjani prescribed it along with marshmallow plant for treatment of patients with pleurisy (22).

Several others plant combinations such as thyme and squill have been recommended for reinforcement of normal breathing and correction of abnormal respiration and treatment of cough, dyspnea, pneumonia and tuberculosis in PM. Each plant may be prescribed based on its mechanism of action and type and cause of dyspnea (such as accumulation of moisture, presence of air (Rih) and retropharyngeal discharge. Avicenna believed that honey can be used in many drug combinations for dyspnea. Despite evidence of anaphylaxis and allergy followed by dyspnea due to the consumption of Ficus Carica, this fruit is recognized as a top home remedy for treatment of asthma (23). This fruit has been used in boiled honey as a basis for many pulmonary medications in traditional medicine.

#### Treatment with manual procedures

The final phase of traditional medicine treatments for elimination of dyspnea involves the use of hands. This method has been recommended after performing the aforementioned two phases unless in particular or emergency cases. Massage of the chest with different oils with and without fabrics is recommended. Type of oil and softness of fabrics used vary depending on the type of disease. Keeping the chest warm is a common recommendation for most patients with shortness of breath. Cupping and bloodletting (if required and if necessary requirements are met) especially at the site of lumbar vertebrae and thighs have also been recommended to alleviate cough and other pulmonary diseases. Phlebotomy as an invasive procedure is only indicated in emergency cases or in patients who did not respond to non-invasive modalities and must be performed in accord with the set regulations. Phlebotomy of the external jugular vein and basilic vein may be suggested in some pulmonary conditions (3).

## CONCLUSION

According to the principles of the Iranian traditional medicine, treatment of shortness of breath requires adherence to specific measures with regard to lifestyle and modifications in type, timing and amount of consumption of beverages and foods, sleep regulation and physical activity. Herbal medicines alone or in combination may be indicated. Recommended medications must have anti-inflammatory effects and must be capable of resolving respiratory tract obstruction by changing the thickness of sputum and affecting airway spasm.

Many of the recommended treatment modalities for dyspnea have long been practiced by traditional medicine specialists and have gained patient satisfaction. By advances in tools and enhanced access to relevant data, we can take advantage of the knowledge of ancient scientists. Non-invasive interventions related to lifestyle modifications are affordable measures with insignificant side effects, which can gain patient satisfaction in many cases.
